# Nano-Nutrition of Chicken Embryos. The Effect of *in Ovo* Administration of Diamond Nanoparticles and l-Glutamine on Molecular Responses in Chicken Embryo Pectoral Muscles

**DOI:** 10.3390/ijms141123033

**Published:** 2013-11-20

**Authors:** Marta Grodzik, Filip Sawosz, Ewa Sawosz, Anna Hotowy, Mateusz Wierzbicki, Marta Kutwin, Sławomir Jaworski, André Chwalibog

**Affiliations:** 1 Division of Nanobiotechnology, Warsaw University of Life Sciences, Ciszewskiego 8, Warsaw 02-786, Poland; E-Mails: marta_grodzik@sggw.pl (M.G.); ewa_sawosz@sggw.pl (E.S.); anna_hotowy@sggw.pl (A.H.); mateusz_wierzbicki@sggw.pl (M.W.); marta_prasek@sggw.pl (M.K.); slawomir_jaworski@sggw.pl (S.J.); 2 Department of Veterinary Clinical and Animal Sciences, University of Copenhagen, Groennegaardsvej 3, 1870 Frederiksberg, Denmark; E-Mail: sawosz@sund.ku.dk

**Keywords:** chicken embryo, myogenesis, diamond nanoparticles, l-glutamine, gene expression

## Abstract

It has been demonstrated that the content of certain amino acids in eggs is not sufficient to fully support embryonic development. One possibility to supply the embryo with extra nutrients and energy is *in ovo* administration of nutrients. Nanoparticles of diamond are highly biocompatible non-toxic carbonic structures, and we hypothesized that bio-complexes of diamond nanoparticles with l-glutamine may affect molecular responses in breast muscle. The objective of the investigation was to evaluate the effect of diamond nanoparticle (ND) and l-glutamine (Gln) on expression of growth and differentiation factors of chicken embryo pectoral muscles. ND, Gln, and Gln/ND solutions (50 mg/L) were injected into fertilized broiler chicken eggs at the beginning of embryogenesis. Muscle tissue was dissected at day 20 of incubation and analysed for gene expression of FGF2, VEGF-A, and MyoD1. ND and especially Gln/ND up-regulated expression of genes related to muscle cell proliferation (FGF2) and differentiation (MyoD1). Furthermore, the ratio between FGF2 and MyoD1 was highest in the Gln/ND group. At the end of embryogenesis, Gln/ND enhanced both proliferation and differentiation of pectoral muscle cells and differentiation dominated over proliferation. These preliminary results suggest that the bio-complex of glutamine and diamond nanoparticles may accelerate growth and maturation of muscle cells.

## 1. Introduction

Modern broiler lines are intensively selected for a higher growth rate and increased size of muscles, including pectoral muscles [1]. This leads to an enhanced requirement of chicken embryos for energy and protein, and consequently the imbalance between requirement and reserves of nutrients stored within eggs may limit maximal (according to genotype) growth and development of chicken embryos. Some authors have indicated that concentrations of certain amino acids in the egg are not sufficient to fully support embryonic development [2,3]. Furthermore, because of limited carbohydrate storage in the eggs, amino acids are important substrates for glycogen synthesis, which may limit their availability for protein synthesis [4]. It has been demonstrated that one possibility to supply embryos with extra nutrients and energy could be *in ovo* nutrition [5,6]. Recently, it has been shown that *in ovo* administration of l-glutamine to chicken embryos increased mRNA and protein level of vascular endothelial growth factor (VEGF-A) [7], which is responsible for endothelial cell proliferation and stimulates vasculogenesis and angiogenesis [8], and affects pectoral muscles morphology [7].

l-glutamine is a key amino acid involved in protein and carbohydrate metabolism. This multifunctional amino acid is a unique provider of amine groups for synthesis of other endogenous amino acids and it is a precursor of proline, which is necessary for formation of connective tissue. Glutamine is also a source of energy, via α-ketoglutarate participation in the Krebs cycle, being an alternative to glucose fuel for rapidly proliferating cells [9]. Furthermore, it is also involved in decreasing excess ammonia from amino acid catabolism, and is also a source of arginine, necessary for uric acid synthesis [10]. Thus, during energy deficiency, l-glutamine can be a consistent source of energy and/or support amino acid synthesis when synthesis of endogenous amino acids is not sufficient for very fast rate of cell proliferation.

Growth and development of muscles are mainly programmed during embryogenesis. The total number of fibers in the pectoral muscle is determined in the prenatal and early post-hatch periods, and not only VEGF-A but also fibroblast growth factor 2 (FGF2) and differentiation factor (MyoD1) are involved in myogenesis. FGF2 is involved in the regulation of muscle growth by stimulation of myoblast and satellite cell proliferation and inhibition of their differentiation. Higher expression of FGF2 during hyperplasia increases the number of the muscle fibers [11]. Opposite to FGF2, MyoD1 activates the process of muscle cell differentiation and inhibits proliferation. Furthermore, expression of myogenic factors increases when the number of myofibers is almost stable, reaching the highest expression on day three in the post-hatched chick [12].

Nutrient supplementation *in ovo* is more efficient when a compound is attached to nanoparticles (silver or gold), which deliver it inside the body tissues and cells [3,7,13]. l-glutamine, when administered to the chicken embryo as a bio-complex of l-glutamine attached to silver nanoparticles, influenced expression of MyoD1 and also enlarged the pectoral muscles in 20-day-old chicken embryos [7]. However, gold and silver are heavy metals, which may be stored within muscles, possibly having toxic effects later in life.

Nanoparticles of diamond, being an allotrope form of carbon with sp^3^ bonds, are highly biocompatible, have an extremely large surface area, high adsorption capacity [14], are easily taken up by cells [15], and are not toxic [14,16,17]. Moreover, they may produce bio-complexes with organic molecules like amino acids by self-organization [18]; hence the bio-complexes can be prepared by simple and fast procedures.

We hypothesized that diamond nanoparticles can affect expression of genes related to embryonic muscle development. Furthermore, these effects can be fortified by administration of bio-complexes of l-glutamine with diamond nanoparticles when glutamine is transported and distributed by nanoparticles, and released into muscle tissue. Thus, the objective of the investigation was to evaluate the effects of diamond nanoparticles, glutamine, and bio-complexes of glutamine conjugated with diamond nanoparticles on the expression of VEGF-A, FGF2, and MyoD1.

## 2. Results

*In ovo* administration of nanoparticles of diamond (ND), l-glutamine (Gln), and the bio-complex of Gln with ND (Gln/ND), evaluated at day 20 of embryogenesis, did not influence the weight of the body, heart, liver, spleen, or pectoral muscle (Table 1). Comparison with the Hamburger-Hamilton normal stages of chicken embryo development [19] showed that all embryos had developed normally. Furthermore, macroscopic evaluation of embryos did not show any genetic or other defects in all embryos.

Biochemical indices measured in the blood serum of chicken embryos were not significantly different, except the concentration of triglycerides, which was lower in the ND and Gln/ND groups compared to the control group (Table 2).

The oxygen consumption at day 10 of incubation was significantly higher in the Gln/ND group compared to the control and Gln groups (Table 3).

Results of expression of *VEGF-A, FGF2*, and *MyoD1* genes normalized to the *β-actin* (*ACTB*) gene on the mRNA level indicated that experimental treatments influenced mRNA synthesis within chicken embryo muscles (Table 4). *VEGF-A* expression was significantly higher in the Gln group than in the control and ND groups. Administration of ND to the embryos significantly increased expression of *FGF2* compared to the control group. Moreover, the bio-complex Gln/ND elevated *FGF2* expression almost twofold, compared to the control group. When *MyoD1* was examined, the level of expression was significantly highest in embryos treated with Gln/ND but also significantly higher in ND than in the control group. The *FGF2:MyoD1* ratio was the highest in the Gln group and the lowest in the Gln/ND group. At the protein level, expression of FGF2 was in line with the mRNA results, being significantly higher for ND and Gln/ND compared to Gln and the control groups. However, there were no significant differences for VEGF-A protein synthesis between measured groups (Table 4).

## 3. Discussion

In the present study experimental solutions were injected into the eggs at day one of incubation and their effects were measured 19 days later. Thus, we assumed that ND and Gln could cross the inner membrane and pass into the developing embryos. This assumption was based on our previous investigations showing that nanoparticles of diamond, platinum, silver, gold and bio-complexes of nanoparticles with amino acids and ATP, when injected at the beginning of incubation, are affecting molecular responses and muscle development measured at the end of embryogenesis [3,7,13,20–23].

The results showed that the hydrocolloid of diamond nanoparticles administered at a concentration of 50 mg/L did not negatively affect chicken embryo growth and development. Furthermore, biochemical indices, measured in the blood serum, did not point to any negative changes within the organism. This result is in agreement with other *in vitro* and *in vivo* investigations of diamond nanoparticles [17,24–26]. However, negative effects of nanoparticles may occur later in the postnatal period, which could be a subject for further research. Embryo growth and development was also not affected by the administration of l-glutamine as well as l-glutamine conjugated with ND. Glutamine is a body component and, used at a low level, should be non-toxic [27].

Although ND, Gln, and Gln/ND did not negatively influence health and growth, there were some effects on the metabolic rate, measured as oxygen consumption, at the beginning of incubation. O_2_ consumption at day 10 of incubation significantly increased in embryos administered Gln/ND. The level of carbohydrate in the egg is very low (2%–3%) and during the first period of embryogenesis, the main energy source is protein [28]. Amino acids may be involved in oxygen-dependent catabolism, and moreover, the key compound providing the Krebs cycle with intermediates is l-glutamic acid, being a precursor of α-ketoglutarate. Thus, it might be suspected that the increased availability of l-glutamine within cell stimulates Krebs cycle turnover and in consequence increases O_2_ consumption. However, in the present experiment, only glutamine attached to diamond nanoparticles significantly increased O_2_ consumption of embryos. It has been shown that small nanoparticles of diamond, less than 50 nm, might penetrate cell membranes and enter inside the cells [29]. Consequently, we could hypothesize that small (1–3 nm) diamond nanoparticles that are resistant to biodegradation might deliver l-glutamine inside the cells and even into the mitochondria where the Krebs cycle takes place.

Considering measurements at a molecular level, the expression of *VEGF-A* at the mRNA level was significantly higher in the Gln group than in the control and ND groups, suggesting pro-angiogenic activities of glutamine [7]. However, when the bio-complex of Gln/ND was applied, the level of *VEGF-A* was not different from the control and ND groups, suggesting that ND may suppress Gln up-regulation of *VEGF-A* as previously demonstrated by Grodzik *et al.* [30]. These observations were not verified at the protein level probably because of the different half-life of mRNA (depending on the oxygen saturation: 4–8 h) [31] and protein VEGF (33.7 ± 13.7 min) [32].

Interesting results were observed regarding *FGF2* and *MyoD1* gene expression. Gln did not increase expression of *FGF2* and *MyoD1* at the mRNA level. In contrast, ND significantly stimulated expression of *FGF2* and *MyoD1* compared to the control group. Studies of other authors revealed the acute responses to ND by increasing the expression of genes responding to oxidative stress and down-regulating expression of genes responding to toxic and genotoxic substances [33]. Furthermore, the activity of ND probably is non-specific and may result from bonding and transporting of signal molecules within the cell or by modifying the local environment. According to Kong *et al.* [34,35], diamond nanoparticles show affinity for protein adsorption, yielding very stable complexes of ND and protein, which may change protein signaling.

Although, ND significantly increased mRNA level of *FGF2* and *MyoD1* compared to the control group, the effect of the bio-complex of Gln/ND was twofold higher than in the control group but also in the ND group, indicating strong abilities of Gln/ND to up-regulate the gene expression. Development of new myocytes depends on a balance between proliferation and differentiation [36]. Signaling of FGF2 is observed from early phases of embryogenesis, mainly being involved in cell proliferation [37–39]. However, the mechanism of increasing the muscle cell number is down-regulated at the end of embryogenesis, but is still active [40], even to a small degree after hatching [41].

Myogenic regulatory factors (including MyoD1) in the embryo muscle are responsible for determination and differentiation of cells, synthesis of myogenin, and suppressing cell proliferation. A high level of proliferation is characteristic for early embryogenesis, and, after reaching a certain number of cells, the process is slowed down and differentiation is initiated [42,43]. Results from the present experiment demonstrated that both “opposite” genes (*FGF2* and *MyoD1*) were strongly activated by Gln/ND treatment, indicating the embryos genetic potential could be utilized to a higher extent, hence the embryos were able to elevate expression of mRNA (*FGF2*, *MyoD1*) and protein (FGF-2) when enriched with l-glutamine delivered by diamond nanoparticles. Moreover, the proportion of mRNA expression of *FGF2* to *MyoD1* was 1:0.92 in the control group, while in the Gln/ND group was 1:1.54. These results, although only based on expression of two genes, suggest that after application of Gln/ND, at the end of embryogenesis differentiation dominates over proliferation. It may be supposed that, at day 20, the pectoral muscle cells were not only activated but also better organized and more mature after administration of Gln/ND.

## 4. Experimental Section

### 4.1. Experimental Design

Broiler chicken eggs from 37-week-old Ross X Ross 308 hens were obtained from a commercial hatchery and stored in a refrigerator (10 °C) for 1–3 days before being placed in an incubator. The eggs were randomly divided into four groups (4 × 40 eggs): without injection (Control), injected with hydrocolloid of diamond nanoparticles (ND), injected with hydrocolloid of l-glutamine (Gln), injected with hydrocolloid of diamond nanoparticles with l-glutamine (Gln/ND). At day 1 of incubation, the eggs were weighed (60 ± 1.36 g) and injected according to the above treatment descriptions. The eggs were injected into the air sac with 0.3 mL of solution using sterile 27-gauge, 20-mm needles. Immediately after the injection, the hole was sealed with sterile tape and the eggs were placed in to the incubator. The eggs were incubated for 20 days under standard conditions (temperature 37.8 °C, humidity 55%, turned once per hour during the first 18 days, at a temperature of 37 °C and humidity 60% from day 19).

At day 20 of incubation, the development status of chicken embryos was compared with the development stages described by Hamburger and Hamilton [19]. Then, the embryos were weighed together with the yolk sack, decapitated, and blood samples from the carotid artery were taken and collected in Eppendorf tubes (Eppendorf AG, Hamburg, Germany). The samples were incubated at room temperature until clotting and then centrifuged for 10 min (14,500 × *g*). The resulting blood serum samples were stored at −30 °C until further analysis. The liver, heart, spleen, and breast muscle were collected and weighed. Samples of the breast muscle for mRNA expression analysis were collected in RNAlater^®^ ribonucleic acid (RNA) stabilization solution (Applied Biosystems/Ambion, Austin, TX, USA), while the samples for protein analysis were frozen at −80 °C.

### 4.2. Solutions

**Figure 1:**
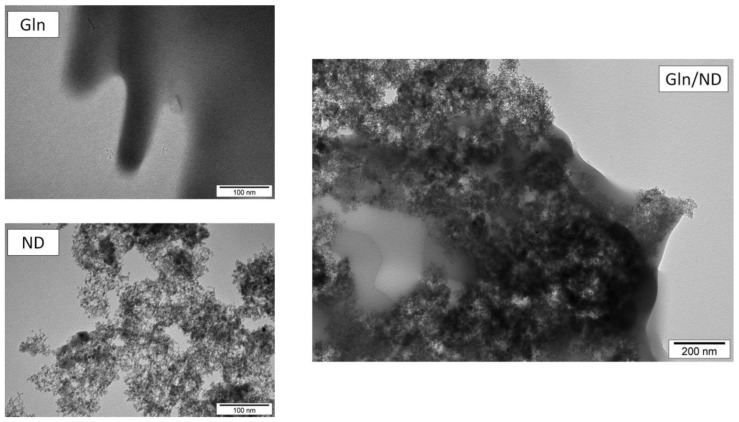
Transmission electron microscopic images of nanoparticles of diamond (ND), l-glutamine (Gln), and bio-complex of Gln with ND (Gln/ND).

Diamond nanoparticles were obtained from Skyspring Nanomaterials (Houston, TX, USA). They were produced by the detonation method with size ranging from 3 to 4 nm. The ND were dispersed in ultra-pure water using sonication at a concentration of 100 mg/L. Pure l-glutamine (Merck, Darmstadt, Germany) was dissolved in ultra-pure water at a concentration of 100 mg/L. A solution of ND conjugated with l-glutamine was prepared by mixed stock solutions using sonication for 30 min at 30 °C in an ultrasonic bath. Concentration of l-glutamine, diamond nanoparticles, and Gln/ND complex in the solutions injected into the eggs was 50 mg/L. Zeta potential of hydrocolloids was examined with a Zetasizer Nano-ZS90 (Malvern Instruments Ltd., Malvern, UK) according to the Wierzbicki method [40]: the zeta potential for ND was −39.3 mV, Gln −10.4 mV, and Gln/ND −38.9 mV. The zeta potential of Gln/ND indicates a stable solution. The shape and bio-complex formation between experimental materials were visualized using a JEM-2000EX transmission electron microscope (JEOL Ltd., Tokyo, Japan) at 200 kV (Figure 1). The picture shows that after mixing solutions of ND and Gln the process of self-organization occurred and the bio-complex of Gln/ND was well established.

### 4.3. Oxygen Consumption

The oxygen consumption was measured at days 10, 13, 16, and 19 of incubation, as previously described [3]. The consumption of O_2_ was measured according to the paramagnetic principle in an open-air-circuit respiration unit (Micro-Oxymax calorimeter, Columbus Instruments, Columbus, OH, USA), equipped with four respiration chambers with a volume of 2000 cm^3^ each. Six eggs from each treatment were placed in each respiration chambers and measured for 3 h from 9:00 to 12:00, followed by another six eggs from the same treatment measured from 13:00 to 16:00. The temperature and relative humidity were kept similar to that in the incubator. After each measurement, the eggs were put back into the incubator. The measurements were standardized to a 50 g egg mass in order to account for differences in weight during each measurement.

### 4.4. Biochemical Indices in the Blood Serum of Chicken

Blood serum concentrations of magnesium, calcium, phosphorus, triglycerides, cholesterol in very-low-density lipoprotein, and glucose, and the activity of alkaline phosphatase, aspartate aminotransferase, alanine aminotransferase, and lactate dehydrogenase were measured by dry chemistry methods using Vitros DT 60 II equipment (Johnson & Johnson, New Brunswick, NJ, USA).

### 4.5. Gene Expression at the mRNA Level

Gene expression at the mRNA level was measured using the quantitative polymerase chain reaction method (qPCR). The tissue dissected from the breast muscle was homogenized in TRIzol^®^ Reagent (Life Technologies, Naerun, Denmark), and total RNA was extracted according to the manufacturer’s instructions. The RNA samples were purified using the SV Total RNA Isolation System (Promega Corporation, Madison, WI, USA) and quantified using a NanoDrop ND 1000 spectrophotometer (Thermo Fisher Scientific, Waltham, MA, USA). Using reverse transcriptase with oligo (dT) (Promega) and random primers (TAG Copenhagen A/S, Copenhagen, Denmark), 2 mg of total RNA was reverse transcribed, after which real-time PCR was performed with complementary DNA and gene-specific primer pairs (TAG, Copenhagen A/S, Copenhagen, Denmark) mixed with LightCycler^®^480 SYBR Green I Master mix (Roche Applied Science, Penzberg, Germany) in a LightCycler^®^ 480 real-time PCR system (Roche Applied Science, Penzberg, Germany). The following primers were used: *FGF2* (forward: GGC ACT GAA ATG TGC AAC AG, reverse: TCC AGG TCC AGT TTT TGG TC), *VEGF-A* (forward: TGA GGG CCT AGA ATG TGT CC, reverse: TCT TTT GAC CCT TCC CCT TT), *MyoD1* (forward: CGG CGG CTC AGC AAG GTC AAC, reverse: CGG CCC GCT GTA CTC CAT CAT G), and *ACTB* (forward: GTC CAC CTT CCA GCA GAT GT, reverse: ATA AAG CCA TGC CAA TCT CG). For each complementary DNA, the reaction was performed in triplicate. For analyses, relative quantification was applied with *ACTB* used as the housekeeping gene. Although, the 2Δ method has been applied, it was possible to use mixed pool of all the samples as a so-called calibrator/positive control, that value was exactly 1.

### 4.6. Gene Expression at the Protein Level

Frozen breast muscle tissue samples were homogenized on ice using RIPA lysis and extraction buffer (Thermo Fisher Scientific, Rockford, IL, USA) and a Polytron^®^ PT 2100 homogenizer (Kinematica AG, Lucerne, Switzerland). Homogenates were left on ice for 30 min and were subsequently centrifuged for 20 min (4 °C, 12,500 rpm). The supernatant was collected in chilled Eppendorf PCR tubes (Eppendorf AG). Supernatant samples were divided into two equal portions. One was used to evaluate the total protein concentration (Total Protein Kit, Micro Lowry, Peterson’s Modification; Sigma-Aldrich, St. Louis, MO, USA). The second portion was used to perform the enzyme-linked immunosorbent test, using an Enzymelinked Immunosorbent Assay Kit (for chicken FGF2, VEGF-A, and ACTB; Uscn Life Science Inc., Wuhan, China). Reagents and plates were prepared accordingly to the manufacturer’s standard procedure and incubated for 25 min under standard conditions. The degree of absorption was measured in a microplate reader Infinite^®^ M200 PRO (Tecan Deutschland GmbH, Crailsheim, Germany) at a wavelength of 450 nm. All samples were measured in duplicate.

### 4.7. Statistical Methods

Analysis of the data was carried out using one-way analysis of variance (ANOVA) procedure of SAS 9.2 (SAS Windows, 2002–2008, version 9.2, SAS Institute Inc., Cary, NC, USA). The Tukey-Kramer honestly significant difference test was used to test separation of the means at a significance level of *p* < 0.05. The results are presented as means and standard errors for each variable.

## 5. Conclusions

We demonstrated that administration of ND, Gln, and Gln/ND at a concentration of 50 mg/L had no negative effects on embryo development. Expression of genes related to proliferation (FGF2) and differentiation (MyoD1) was up-regulated by ND and especially Gln/ND. Furthermore, the ratio between FGF2 and MyoD1 was highest in the Gln/ND group, suggesting that at the end of embryogenesis, Gln/ND enhanced both proliferation and differentiation of pectoral muscle cells and that differentiation dominated over proliferation. These preliminary results suggest that the bio-complex of glutamine and diamond nanoparticles may accelerate growth and maturation of muscle cells.

## Figures and Tables

**Table 1:** Average weight of chicken embryos, organs, and pectoral muscles in the control group and groups treated with nanoparticles of diamond (ND), l-glutamine (Gln), and bio-complex of Gln with ND (Gln/ND), *n* = 40/group.

	Groups				ANOVA	
	
	Control	ND	Gln	Gln/ND	SEM	*p*-Value
Embryo [% e.w.]	78.7	77.0	76.2	76.4	4.183	NS
Heart [% b.w.]	0.46	0.42	0.47	0.44	0.032	NS
Liver [% b.w.]	1.42	1.42	1.36	1.40	0.181	NS
Spleen [% b.w.]	4.03	3.81	3.37	3.81	0.674	NS
Muscle [% b.w.]	0.84	0.97	0.80	0.88	0.011	NS

e.w. = egg weight; b.w. = body weight; NS = non-significant, *p* < 0.05.

**Table 2:** Biochemical indices in the blood serum of 20-day-old chicken embryos in the control group and groups treated with nanoparticles of diamond (ND), l-glutamine (Gln), and bio-complex of Gln with ND (Gln/ND), *n* = 40/group.

	Groups				ANOVA	
	
	Control	ND	Gln	Gln/ND	SEM	*p*-Value
Magnesium [mmol/L]	0.87	0.87	0.91	0.88	0.043	NS
Calcium [mmol/L]	2.25	2.15	2.13	2.14	0.178	NS
Phosphate [mmol/L]	1.31	1.29	1.42	1.40	0.082	NS
Triglycerides [mmol/L]	1.04 A	0.65 B	0.83 AB	0.76 B	0.072	0.01
Cholesterol [mmol/L]	11.23	9.35	10.48	9.28	1.554	NS
Glucose [mmol/L]	14.9	14.5	13.5	14.0	0.651	NS
Alkaline phosphatase [U/L]	6125	7078	7023	6917	970.74	NS
Aspartate aminotransferase [U/L]	179	153	126	151	22.91	NS
Alanine aminotransferase [U/L]	3.67	3.33	2.00	3.33	1.001	NS
Lactate dehydrogenase [U/L]	1778	1564	1360	1612	346.64	NS

NS = non-significant;

A, B, AB —within rows, means bearing different superscripts differ significantly at *p* < 0.05.

**Table 3:** Oxygen consumption (O_2_) in the control group and groups treated with nanoparticles of diamond (ND), l-glutamine (Gln), and bio-complex of Gln with ND (Gln/ND), *n* = 40/group.

	Groups				ANOVA	
	
	Control	ND	Gln	Gln/ND	SEM	*p*-Value
O_2_ [mL/h]						
10 ED	2.91 A	3.81 AB	3.02 A	4.21 B	0.473	0.02
13 ED	10.9	10.3	10.3	11.8	1.132	NS
16 ED	25.9	25.1	25.9	23.1	2.044	NS
19 ED	32.8	31.4	31.5	30.4	2.491	NS

NS = non-significant; ED = embryonic day;

A, B, AB —within rows, means bearing different superscripts differ significantly at *p* < 0.05.

**Table 4:** Gene expression in the breast muscle tissue of chicken embryos in the control group and groups treated with nanoparticles of diamond (ND), l-glutamine (Gln), and bio-complex of Gln with ND (Gln/ND) at the mRNA and protein level. All results (mRNA and protein) were normalized to ACTB, *n* = 40/group.

	Groups				ANOVA	
	
	Control	ND	Gln	Gln/ND	SEM	*p*-Value
**mRNA level:**						
*VEGF-A/ACTB*	1.24 A	1.17 A	1.61 B	1.38 AB	0.19	0.02
*FGF2/ACTB*	0.74 A	0.90 B	0.85 AB	1.50 C	0.08	0.00
*MyoD1/ACTB*	0.68 A	1.19 B	0.62 A	2.31 C	0.23	0.00
*FGF2:MyoD1* ratio	1:0.92	1:1.32	1:0.73	1:1.54		
**protein level:**						
VEGF-A/ACTB	1.24	1.17	1.03	0.05	0.15	NS
FGF2/ACTB	0.73 A	0.87 B	0.75 A	0.93 B	0.06	0.00

NS = non-significant;

A, B, AB, C —within rows, means bearing different superscripts differ significantly at *p* < 0.05.
